# Impact of Exergaming on Children’s Motor Skill Competence and Health-Related Fitness: A Quasi-Experimental Study

**DOI:** 10.3390/jcm7090261

**Published:** 2018-09-07

**Authors:** Sunyue Ye, Jung Eun Lee, David F. Stodden, Zan Gao

**Affiliations:** 1 Department of Sports Rehabilitation, College of Physical Education, Longyan University, Longyan 364012, China; 2 Chronic Disease Research Institute, School of Public Health, School of Medicine, Zhejiang University, Hangzhou 310058, China; 3 Department of Applied Human Sciences, University of Minnesota, Duluth, MN 55812, USA; junelee@d.umn.edu; 4 Department of Physical Education, University of South Carolina, Columbia, SC 29208, USA; stodden@mailbox.sc.edu; 5 School of Kinesiology, University of Minnesota, Minneapolis, MN 55455, USA

**Keywords:** active video games, cardiorespiratory fitness, locomotor skills, motor skill competence, musculoskeletal fitness, object control skills

## Abstract

This study was designed to examine the effectiveness of a combined exergaming and physical education (PE) program on children’s motor skill competence (MSC) and health-related fitness (HRF) as compared to traditional PE. A total of 261 second- and third-grade children (127 boys; 8.25 ± 0.66 years for male; 8.29 ± 0.74 years for female; 73.6% non-Hispanic white) participated in the nine-month study from 2012 to 2013. Children were assigned to one of the two groups: (a) intervention group (125 min of alternating PE and exergaming weekly); and (b) comparison group (125-min weekly PE). MSC was assessed via product scores in two locomotor and two object control skills. HRF included the cardiorespiratory fitness, musculoskeletal fitness, and body mass index (BMI). A multivariate analysis of variance (MANOVA) was performed to analyze the effect of the combined exergaming–PE program on children’s MSC and HRF. There were significant group by time interaction effects for BMI, *p* < 0.01, *η*^2^ = 0.20; musculoskeletal fitness, *p* < 0.01, *η*^2^ = 0.13; and object control skills (the comparison group demonstrating greater improvement), *p* = 0.01, *η*^2^ = 0.03. The findings suggest that the combined exergaming program can have a positive effect on children’s BMI and musculoskeletal fitness, indicating that exergaming can be an alternative school-based program to supplement traditional PE.

## 1. Introduction

Secular declines in children’s health-related fitness (HRF) is a growing concern globally [1,2,3] and places children at risk for many health issues, including cardiovascular diseases or metabolic syndrome [4], obesity [5], and lower health-related quality of life [6,7,8]. Similarly, inadequate levels of motor skill competence (MSC) are a concern among health professionals and physical educators [9,10] as the development of MSC is positively linked to children’s HRF [11,12] and physical activity (PA) [12,13]. Specifically, competence in fundamental locomotor (e.g., jumping and hopping) and object control (e.g., throwing and kicking) skills is a prerequisite to the development of transitional movement skills [10]. MSC can be enhanced at an early age through targeted intervention, which also is beneficial for overall HRF [14].

Although a body of literature has shown traditional school-based PA programs or physical education (PE) courses can be effective in improving children’s HRF [15] and MSC [16,17] in the short term, interventions with long-term sustainability are still needed [15]. To sustain development in children’s MSC and HRF, it is critical to design an integrated and fun PA intervention that appeals to children [18]. Exergaming (a.k.a., active video games), a type of video games that requires bodily movement to play the video game, has been shown to increase children’s light-to-moderate PA by capitalizing on their interests in games and maintaining PA enjoyment [9,19,20,21]. Exergaming is one of the innovative and fun ways to motivate children to be active and develop their motor skills [20,21,22]. For example, exergaming allows children of all skill levels to engage in sport games that they would not be able to unless they have the appropriate skill levels in traditional PE classes. This is also possible because in exergaming, children can still play without needing appropriate equipment for specific sports. A few studies have indicated that integrating exergaming into PE classes contributes to higher levels of children’s PA and energy expenditure in both the short- and long-term, possibly due to its fun components [23,24,25,26]. While exergaming is an effective means to promote children’s PA, evidence on school-based exergaming interventions to promote MSC is lacking [23,27].

Recent studies demonstrated that exergaming has an inconsistent impact on children’s MSC in the short term [28,29]. Zeng and Gao, in 2016, concluded in a systematic review that studies that prove exergaming can offer sufficient stimulus for MSC changes were still limited [30]. Moreover, most of these studies examined balance skills or postural stability, with very few addressing the two important categories of MSC in young children [30,31,32]. Furthermore, no studies have explored the combined effects of exergaming and PE on young children’s MSC and HRF across a nine-month school-based intervention. Therefore, this quasi-experimental study was designed to examine the effectiveness of school-based exergaming integration with traditional PE on children’s MSC and HRF compared to traditional PE.

## 2. Materials and Methods

### 2.1. Participants and Research Setting

A total of 261 second- and third-grade children (8.27 ± 0.70 years; 134 girls; 73.6% non-Hispanic white) from two public elementary schools in southern USA participated in this study across the school year. Due to administrative and logistical reasons, randomization of individual children into either the intervention group or the comparison group was not feasible. Hence, one school served as the intervention school with the other serving as the comparison group. Both public schools were Title I schools (i.e., more than 50% children receive free or reduced-price school meals) in the same school district and were similar in their demographics. The majority of children were from low-income families. Other detailed information about the participants and research setting can be found in a recently published paper [23]. The inclusion criteria for this study were children who were (1) enrolled in a public Title I elementary school; (2) aged 7–9 years; (3) without a diagnosed physical or mental disability according to school records; and (4) able to provide parental consent and child assent. The eligibility of inclusion was verified through demographic information and student records in the schools.

**Figure 1:**
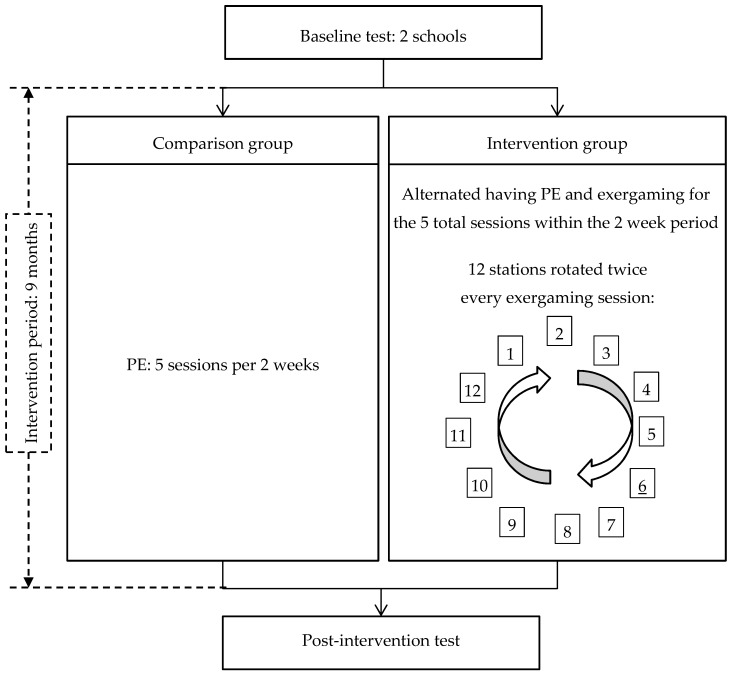
Study design and intervention flow.

In the intervention school, the exergaming program was integrated into the school’s PE curriculum in such a way that exergaming and PE days alternated each week for a combined total of 125 min of biweekly PA; that is, out of 5 days of 25 min classes every two weeks, children’s schedules alternated having 3 PE classes in one week and 2 exergaming sessions the following week (Figure 1). A certified PE teacher taught PE and a full-time teacher, trained by the research team, coordinated and supervised children in the exergaming room (i.e., a modified classroom) at the school. In the comparison school, children had 125 min (25 min per class) of PE every two weeks taught by 2 certified teachers. Participants were recruited from 16 classes (average of ≈20 students per class) at the schools, with 8 classes from each school site. The study was approved by the University Institutional Review Board and parental consent and child assent were obtained.

### 2.2. Procedures

In the intervention school, 12 stations (costing approximately 600 US dollars per station), each equipped with 2 exergaming systems including Wii (Nintendo Co., Ltd., Kyoto, Japan) and Xbox Kinect (Microsoft Corp., Redmond, WA, USA) and a television, were installed in a classroom. Examples of the types of exergaming included were Kinect Ultimate Sports, Just Dance, Wii Sports, and Wii Fit. The variety of games allowed for autonomy in selection and promoted persistent motivation throughout the whole intervention period. A trained teacher supervised their participation of exergaming. Depending on the type of game, either all children participated in an exergaming session simultaneously (i.e., two children/station) or all children participated in one activity (e.g., Just Dance). Children rotated games twice during each session. During PE classes at both schools, a conventional multiactivity curriculum was promoted that mainly included sport- or game-based units and fitness activities [33].

### 2.3. Measures

Participants demographic information including age and gender was collected using an information sheet filled in by students, with assistance from research staff before intervention.

MSC assessments included performance on four skills (kicking, throwing, standing long jump, and hopping) based on our and other experts’ studies [34,35,36,37,38]. We used performance scores of skills to measure children’s MSC because the measures are sensitive in discriminating children’s competence levels across childhood and align with validated process-oriented assessments of motor skills [34,35,36,37]. The maximum speed for kicking (20-cm diameter playground balls) and throwing (using tennis balls) and maximum standing long jump distance were assessed from a total of five trials and used for analysis [38]. Throwing and kicking speed was measured using a radar gun (Stalker Radar, Plano, TX) [38]. For hopping, the average height of a minimum of three hops for each leg was used for analysis [35]. Both standing long jump and hopping performance were calculated based on children’s standing height [34,35]. The reliability of these tests (alpha coefficient method) were r = 0.70 (*p* < 0.01) according to a previous study [10].

Cardiorespiratory fitness and musculoskeletal fitness components of children’s HRF was measured using the *FITNESSGRAM*^®^. Trained research staff implemented *FITNESSGRAM*^®^ protocols for the Progressive Aerobic Cardiovascular Endurance Run (PACER), curl-ups, and push-ups [38]. Grip strength was also assessed using a children’s grip dynamometer (Lafayette Instrument, Lafayette IN) as an additional indicator of musculoskeletal fitness [39]. The best score of three trials for each hand was averaged and used for data analysis [23]. To assess body composition component of HRF, children’s body mass index (BMI) was calculated as the weight in kilograms divided by the height in meters squared. Children’s body height and weight were measured using a stadiometer (Seca GmbH, Hamburg, Germany) and digital weight scale (Detecto, Web City, MO, USA), respectively. The validity and reliability of all of these measures have been noted in previous studies [34,35,38,39,40]. Baseline MSC and HRF tests were measured in September of 2012 and post-tests were conducted in May of 2013.

### 2.4. Data Analysis

The demographic characteristics of participants were described through a descriptive analysis. Missing data of one or some indices of MSC and HRF were imputed by the expectation maximization (EM) method, although participants with missing data for all indices were excluded. All indices of MSC and HRF scores were normalized and then converted to standard T-scores (T-score = 50 + 10 * Z-score). Performance scores for the MSC tests were grouped according to locomotor and object control separately. The locomotor skill score was the mean T-scores for both standing long jump and hopping, while object control skill score was defined as the mean score of kicking and throwing T-scores. In regard to HRF, the musculoskeletal fitness score was calculated as the mean of the T-scores of grip strength, push-ups, and curl-ups.

T-tests and paired T-tests were conducted to observe any differences in all variables of interest between the comparison and intervention groups and between the pre- and post-intervention tests, respectively. Since there were significant between-group differences in baseline scores of PACER (higher in comparison group) and BMI (higher in intervention group), as well as curl-up (higher in comparison group), the difference in scores between pre- and post-tests for each variable were set as the outcome variable. In order to explore the effects of intervention on children’s MSC and HRF over time, a multivariate analysis of variance (MANOVA) was conducted. All descriptive and inferential statistical analyses were conducted using SPSS 20.0 (IBM Corp., Armonk, NY, USA) and the significance level was set at 0.05 for all statistical analyses.

## 3. Results

Eleven children were removed from the study from pre-test to post-test due to missing data for all indices of MSC and HRF (*n* = 10) or being an outlier (*n* = 1). Baseline demographic characteristics of participants are shown in Table 1. Children’s mean age was 8.27 years, and 75% of the sample was non-Hispanic white. The intervention effects on children’s MSC and HRF are described in Table 2. Significant group by time interaction effects were revealed for HRF and MSC variables including: musculoskeletal fitness, *F* (1, 250) = 38.33, *p* < 0.01, *η*^2^ = 0.13; BMI, *F* (1, 250) = 61.39, *p* < 0.01, *η*^2^ = 0.20; and object control skills, *F* (1, 250) = 6.77, *p* = 0.01, *η*^2^ = 0.03. While children in the comparison group displayed significantly higher cardiorespiratory fitness scores (PACER) than children in the intervention group at both baseline and post-tests (*p* < 0.01), the comparison and intervention groups both demonstrated increase over time in their cardiorespiratory fitness (*p* < 0.01). Likewise, the decline in intervention children’s BMI demonstrated a significant difference from the increased BMI in the comparison group. In addition, the between-group difference in the change scores of children’s musculoskeletal fitness was significant, favoring the intervention group.

In terms of children’s MSC, significant improvement effects over time were revealed in children’s object control skills in both the intervention and comparison groups (*p* < 0.01), with the comparison group demonstrating greater improvement, *F* (1, 250) = 6.77, *p* = 0.01, *η*^2^ = 0.03, compared to the intervention group. Slight improvement in the locomotor skills of children was observed in the intervention and comparison groups over time; however, these changes in score were not significant.

## 4. Discussion

As an innovative technology and potential tool for capturing and maintaining children’s interest and motivation to be physically active and fit [41], exergaming has been integrated into a traditional PE curriculum within the school day [24,25]. However, the impact of exergaming on children’s motor skills is not well understood. Our findings indicate that an integrated school-based exergaming/PE program demonstrated greater overall positive benefits on children’s muscle strength and BMI, compared to traditional PE only. This impact on fitness may be due to: (1) more light and moderate PA in exergaming compared to fitness activities offered in PE classes, which could lead to body weight improvement in children [42], and (2) specificity of fitness exergames (i.e., Wii Fit and Just Dance) offered in the intervention group, which can improve children’s some musculoskeletal fitness. Playing games from the Wii system may have improved children’s grip strength through use of the controllers [43]. Previous evidence has illustrated controversy on the notion that exergaming provides sufficient PA intensity to improve children’s HRF [19]. Specifically, some literature did not support the capability of exergaming to improve children’s physical fitness or to provide health benefits [19,27,44,45], while other literature suggests exergaming can promote health benefits associated with maintenance of weight status and increased cardiorespiratory fitness [46,47]. The inconsistent findings might be owed to the setting in which exergames were played (e.g., laboratory, home, or school), types of the games, duration/dose, and intervention fidelity [48,49,50]. Previous studies have shown that the following factors are important in enhancing children’s motivation, ultimately leading to appropriate improvement in their HRF: (a) presence of the supervision of specialist teachers; (b) appropriate organization behavior management; and (c) including a variety of PA programs [16,51,52]. Thus, we speculate that adequate supervision and consistent incorporation of different types of exergames offered to children led to greater increases in musculoskeletal fitness, as most of the previous relevant studies have intervened through only one type of exergame [20,49,53,54].

Our findings in children’s MSC only partially supported the other portion of our hypothesis. When examining locomotor skills data, there was no significant difference in changes of score between the comparison and the intervention group, suggesting little impact of exergames on children’s hopping and standing long jump. While the comparison group demonstrated significantly greater improvement in object control skills as compared to the intervention group across the school year, the effect size was small (*η*^2^ = 0.03). The significant increase in object control skills in the traditional PE groups may be indicative of having more overall time spent in performing object control skills in a more ecologically valid setting (e.g., more time spent in PE). Specifically, no Wii or Xbox games provide opportunities for actual kicking and throwing activities. Games noted within exergaming games (e.g., Wii Sports and Kinect Ultimate Sports) do not necessarily demand that individuals demonstrate their highest level of throwing (e.g., primarily using elbow extension and wrist flexion in the Wii baseball game). Thus, the virtual games promoted in exergaming may not demand the same type of movement execution and effort to promote skill development to the same degree as traditional practice experiences.

In the present study, kicking skills of children in the intervention group increased over time; however, their standing long jump decreased at the end of the program. It appears that development of the standing long jump (locomotor skills) through semi-structured exergaming is more challenging than acquiring kicking skills (object control skills). Indeed, in two recent studies [31,55], similar findings were shown. The researchers tested the efficacy of six 50-min sessions of active video games (AVGs) on 6–10-year-olds’ actual and perceived objective motor skills. Not only did they not find any significant differences between the control and intervention groups on both outcomes, they also did not find any improvement in the skills between typically developing children and children with autism spectrum disorder. The authors suggested that the play nature of AVGs may not provide adequate practice of the correct movements that are required to perform the skills [31,55]. Similarly, Pedersen et al., in their study in which they examined the effects of 30-min Nintendo Wii tennis games in improving reaction time for contralateral arm movement in children aged 7–12 years, revealed that these short bouts of exergaming sessions did not enhance children’s motor processing speed (reaction time) [56]. Reaction time may not be directly related to the performance of object control skills measured in the current study; however, it is a good indicator of how children’s object control skills would be applied in more advanced sport skill settings.

On the contrary, other studies have shown that the use of exergaming could be a feasible and pleasant approach to improve elementary school or nontypically-developing children’s gross motor skills [32,57]. In one of these studies, an eight-week long exergaming intervention was found effective in improving children’s object control skills. In this case, however, the effectiveness of the intervention in enhancing the skills was due to the selection and implementation of exergames (i.e., Baseball mini games; NBA Baller Beats, Bowling, and Soccer mini games; Xbox Kinect) that were specifically geared toward developing the same six objective control skills, namely throwing, kicking, catching, dribbling, rolling, and striking. Based on these findings of previous literature, we speculate that the lack of games that would provide opportunities for children to practice throwing and kicking skills could have been one of the reasons why we did not observe improvement in object control skills in the current study.

Another important point to note may be the quality of play, to which Howie and her colleagues attributed the ineffectiveness of an exergaming (Xbox 360 with Kinect) intervention on children’s motor coordination (MABC-2) [58]. In this home-based exergaming intervention study for the clinical pediatric population, the authors mention motivation–outcome trade-off, which describes how self-selected games may increase children’s motivation to play longer, but not necessarily the quality of play that leads to motor skill improvement. The study suggests the importance of finding a challenging point for each individual and adhering to the game to experience motor skill improvement, instead of switching games that they favor. We speculate that this may be very much aligned to our findings in current study. Children in this present study could have played longer and at higher intensities to increase their HRF; however, not at high enough quality to contribute to improvement in object control skills.

In our previous study, in which we examined the effect of an exergaming program in increasing children’s PA, we did not see any significant increase in their PA levels [23]. However, significant exergaming effects on children’s HRF were observed in our current study. It is plausible that these inconsistent results between the two studies are due to several possible reasons. First, measurement of children’s PA was limited to school time, not whole days (24 h), which could not reflect actual total levels of daily PA. Second, increase in children’s HRF may not be due to total PA levels, but more due to other factors such as dietary intake, basic metabolic rate, and intensity of exercise. Third, it is possible that exergaming had a beneficial effect in current study because it was combined with PE. In this case, PE can provide opportunities that exergaming cannot offer and lead to greater effects of intervention than the effects that would be brought by PE or exergaming alone. However, further studies are warranted to explore the potential interactions.

This study was conducted in a real-world setting, examining the effects of a combination of exergaming and traditional PE, and including both HRF and MSC as outcome variables, which are the strengths of this study. However, the study is not without limitations. First, our study did not implement random recruitment or random assignment, which limits the generalizability. Second, it is also difficult to define the independent effect of exergaming on the outcomes as our intervention had both PE and exergaming sessions. Third, flexibility, which is one of the five HRF components, was not included in our HRF outcomes, and this study could improve by implementing a follow-up measurement to avoid potential seasonal effects on children’s HRF/MSC and to explore the durability of children’s interest in exergaming. Moreover, children in our study could have been exposed to a “Hawthorne Effect”, as teachers and research assistants were observing them at the time of testing. Children could have modified their behavior (e.g., making much more effort in physical activity) in response to their awareness of being observed or intervened. In addition, there might have been some motivational issues in children in the comparison group at the curl-ups testing, as the post-test score plummeted from the baseline. Examining our data, a good number of children who scored high in the baseline did not do as well in their post-test, which might have influenced the overall musculoskeletal fitness score in the comparison group. Lastly, the information about energy expenditure was not collected during the exergaming and PE sessions.

## 5. Conclusions

A well-implemented class combining exergaming and traditional PE can be beneficial in developing children’s musculoskeletal fitness and BMI compared to the sole traditional PE class. Considering the potential benefits of exergaming in stimulating children’s interest in PA and developing HRF [59], integration of an exergaming component into PE is partly recommended. However, when exergaming is implemented, several things should be taken into consideration. The structure of the exergaming may depend on the goal of the program, whether it be promotion of PA, enhancement of HRF, or/and improvement in MSC. If practitioners are in pursuit of children’s MSC, the traditional PE classes may be a better choice than exergaming. Future studies with randomization and long-term design are needed to confirm our results.

## Figures and Tables

**Table 1:** Demographic characteristics of participants in baseline and post-intervention.

Variables *	Baseline			Post-Intervention		
	Comparison (*n* = 115)	Intervention (*n* = 135)	*p* Value ^‡^	Comparison (*n* = 115)	Intervention (*n* = 135)	*p* Value ^‡^
Age (years)	8.41 (0.71)	8.14 (0.67)	0.002	/	/	/
Girls (counts) ^†^	59 (51.3)	70 (51.9)	0.931	/	/	/
White American (counts) ^†^	69 (60.0)	118 (87.4)	<0.001	/	/	/
Height (cm)	131.77 (7.59)	129.46 (6.83)	0.012	132.73 (7.24)	132.80 (7.19)	0.942
Weight (kg)	30.77 (9.12)	31.69 (8.69)	0.417	33.73 (9.65)	32.95 (9.72)	0.524
Motor skill competence						
Kicking (m/s)	10.95 (1.79)	11.06 (2.46)	0.691	12.06 (1.87)	11.84 (1.92)	0.360
Throwing (m/s)	14.10 (3.82)	14.55 (3.35)	0.317	14.74 (3.87)	14.48 (3.69)	0.586
Standing long jump (%)	0.93 (0.15)	0.93 (0.17)	0.979	0.93 (0.15)	0.91 (0.17)	0.390
Hops (%)	0.64 (0.12)	0.62 (0.14)	0.190	0.67 (0.12)	0.70 (0.14)	0.124
Health-related fitness						
PACER (laps)	22.92 (11.15)	14.04 (7.08)	<0.001	26.87 (13.24)	20.67 (9.30)	<0.001
Grip strength (kg)	13.90 (2.96)	14.50 (3.48)	0.143	12.86 (3.66)	16.22 (4.01)	<0.001
Push-ups (counts)	7.47 (6.26)	6.24 (6.07)	0.116	9.32 (6.47)	8.65 (6.23)	0.409
Curl-ups (counts)	34.60 (26.57)	23.49 (21.86)	<0.001	18.58 (18.93)	21.33 (19.65)	0.262
BMI (kg/cm^2^)	17.60 (3.78)	18.72 (3.67)	0.019	18.88 (3.89)	18.42 (3.87)	0.346

Note: * All values are the mean, with standard deviation in brackets; ^†^ Frequencies (percentage); ^‡^ Student’s T-test for continuous variables and chi-squared test for categorical variables; Standing long jump (%): standing long jump (cm) divided by body height (cm); Hops (%): hops (cm) divided by body height (cm); PACER: progressive aerobic cardiovascular endurance run; BMI: body mass index.

**Table 2:** Baseline and post-intervention motor skill competence and health-related fitness descriptive and inferential statistics.

Tests	Conditions	Baseline T-Scored Mean (SD)	Post-Test T-Scored Mean (SD)	Diff. Mean (SD)	F	*p*	*η* ^2^
*MSC*							
Locomotor	Intervention (*n* = 135)	49.34 (7.23)	50.11 (7.21)	0.77 (4.64)	1.03	0.311	0.00
	Comparison (*n* = 115)	49.77 (6.68)	49.98 (6.68)	0.20 (4.16)			
Object control	Intervention (*n* = 135)	49.02 (9.21)	50.87 ^‡^ (9.01)	1.85 (5.51)	6.77	0.010	0.03
	Comparison (*n* = 115)	48.20 (8.45)	51.73 ^‡^ (8.66)	3.54 (4.59)			
*HRF*							
PACER	Intervention (*n* = 135)	44.23 ^†^ (6.33)	49.00 ^‡^ (8.56)	4.77 (6.40)	1.77	0.184	0.01
	Comparison (*n* = 115)	52.21 (9.95)	55.69 ^‡^ (11.84)	3.48 (8.93)			
Musculoskeletal fitness	Intervention (*n* = 135)	49.37 (6.74)	51.25 ^‡^ (7.08)	1.88 (5.19)	38.33	<0.001	0.13
	Comparison (*n* = 115)	50.83 (7.64)	48.30 ^‡^ (6.08)	−2.53 (6.08)			
Body mass index	Intervention (*n* = 135)	50.81 * (9.76)	50.01 ^†^ (10.31)	−0.80 (4.33)	61.39	<0.001	0.20
	Comparison (*n* = 115)	47.83 (10.06)	51.24 ^‡^ (10.35)	3.41 (4.12)			

Note: Musculoskeletal fitness: represents the compiled score for grip strength, curl-ups, and push-ups tests; SD: standard deviation; MSC: motor skill competence; Locomotor: combining standing long jump and hops; Object control: combining kicking and throwing; HRF: health-related fitness; PACER: progressive aerobic cardiovascular endurance run; Musculoskeletal fitness: combining grip strength, push-ups, and curl-ups; Diff.: difference between the baseline and post-test. T test was conducted to observe the difference between comparison and intervention groups at the baseline. * *p* < 0.05 compared with comparison group; ^†^ *p* < 0.01 compared with comparison group; ^‡^ *p* < 0.01 compared with baseline.
